# A pragmatic randomised controlled trial (RCT) and realist evaluation of the interdisciplinary home-bAsed Reablement program (I-HARP) for improving functional independence of community dwelling older people with dementia: an effectiveness-implementation hybrid design

**DOI:** 10.1186/s12877-019-1216-x

**Published:** 2019-07-29

**Authors:** Yun-Hee Jeon, Judy M. Simpson, Lee-Fay Low, Robert Woods, Richard Norman, Loren Mowszowski, Lindy Clemson, Sharon L. Naismith, Henry Brodaty, Sarah Hilmer, Amanda Miller Amberber, Laura N. Gitlin, Sarah Szanton

**Affiliations:** 1 Susan Wakil School of Nursing and Midwifery, The University of Sydney, 88 Mallett Street – Building M02, Camperdown, Sydney, NSW Australia; 2 Sydney School of Public Health, The University of Sydney, Camperdown, Australia; 3 Faculty of Health Sciences, The University of Sydney, Sydney, Australia; 4 Bangor University, Bangor, UK; 5 Curtin University, Perth, Australia; 6 Brain and Mind Centre and School of Psychology, The University of Sydney, Sydney, Australia; 7 Brain and Mind Centre, Charles Perkins Centre and School of Psychology, The University of Sydney, Sydney, Australia; 8 CHeBA (Centre for Healthy Brain Ageing), School of Psychiatry, UNSW, Sydney, Australia; 9 Kolling Institute, Northern Clinical School, Faculty of Medicine and Health, University of Sydney and Royal North Shore Hospital, St Leonards, NSW Australia; 10 Charles Sturt University, Albury Wodonga, Australia; 11 College of Nursing and Health Professions, Drexel University, Philadelphia, PA USA; 12 Johns Hopkins School of Nursing, Johns Hopkins School of Public Health, Baltimore, MD USA

**Keywords:** Dementia, Interdisciplinary teamwork, Community care, Reablement, Cognitive rehabilitation, Pragmatic trial, Implementation

## Abstract

**Background:**

A major gap exists internationally in providing support to maintain functional and social independence of older people with dementia living at home. This project evaluates a model of care that integrates evidence-based strategies into a person-centred interdisciplinary rehabilitation package: Interdisciplinary Home-bAsed Reablement Program (I-HARP). Two central aims are: 1) to determine the effectiveness of I-HARP on functional independence, mobility, quality of life and depression among people with dementia, their home environmental safety, carer burden and quality of life, and I-HARP cost-effectiveness; and 2) to evaluate the processes, outcomes and influencing factors of the I-HARP implementation.

**Methods:**

I-HARP is a 4-month model of care, integrated in community aged care services and hospital-based community geriatric services, and consists of: 1) 8–12 home visits, tailored to the individual client’s needs, by an occupational therapist, registered nurse, and other allied health staff; 2) minor home modifications/assistive devices to the value of <A$1000 per participant; and 3) three individual carer support sessions. The overarching design is a mixed-methods action research approach, consisting of a multi-centre pragmatic parallel-arm randomised controlled trial (RCT) and realist evaluation, conducted in two phases. Participants include 176 dyads (person aged > 60 years with mild to moderate dementia and his/her carer). During Phase I, I-HARP advisory group is established and training of I-HARP interventionists is completed, and the effectiveness of I-HARP is examined using a pragmatic RCT. Phase II, conducted concurrently with Phase I, focuses on the process evaluation of the I-HARP implementation using a realist approach. Semi-structured interviews with participants and focus groups with I-HARP interventionists and participating site managers will provide insights into the contexts, mechanisms and outcomes of I-HARP.

**Discussion:**

I-HARP is being evaluated within the real-world systems of hospital-based and community-based aged care services in Australia. Future directions and strategies for reablement approaches to care for community dwelling people living with dementia, will be developed. The study will provide evidence to inform key stakeholders in their decision making and the use/delivery of the program, as well as influence future systems-thinking and changes for dementia care.

**Trial registration:**

Australian New Zealand Clinical Trial Registry ACTR N12618000600246 (approved 18/04/2018).

## Background

One of the hallmarks of dementia and its progression is decline in the person’s capacity to self-care and maintain independence, often expressed as functional impairment in Activities of Daily Living (ADL): both basic (e.g. grooming, feeding and toileting) and instrumental (e.g. managing finances, problem-solving, handling medication, and housekeeping) [1, 2]. Without appropriate care and support, these impairments have detrimental impacts on the person’s physical, social and psychological health, and quality of life. However, functional decline may not be solely caused by the neurodegenerative nature of dementia. In fact, functional decline may be age-related, or associated with other chronic illness, and exacerbated by their physical or social environment [3].

People with dementia are more likely to be hospitalised and have worse outcomes during hospitalisations than those without dementia, including a higher chance of developing harmful outcomes or preventable complications [4, 5]. They also have a high prevalence of physical and medical comorbidities, such as stroke, diabetes and visual impairments, falls, delirium, weight loss, malnutrition, epilepsy, frailty, sleep disorders, oral disease, and incontinence [6–8]. A recent Swedish population-based cohort study reports that people with dementia experienced multimorbidity more often than those without, and dementia combined with multimorbidity was associated with faster functional decline, 1–2 years ahead of those with dementia but without any co-morbidity [8]. With or without dementia, multimorbidity is highly prevalent in old age. More than a decade ago, a Canadian study showed that 98% of older people in primary care had multimorbidity [9]; this is a continuing and increasing trend, according to the latest Global Burden of Disease Study from 2013 [10].

Approximately two thirds of Australians with dementia live at home [11], and most of them have multimorbidity with long-term and complex care needs. A large and growing proportion of community aged care services cares for people with dementia. The person with dementia’s ability to self-care and live independently with some support from others is one of the most important factors influencing their capacity to remain at home and their quality of life. The Clinical Practice Guidelines and Principles of Care for People with Dementia [12] Practice Point 66 states that care “*… should aim to promote and maintain functional and social independence of people with dementia in community and residential care settings … should address activities of daily living that maximise independence, function and engagement”*. Thus, there is a critical need to develop and evaluate community care service models rooted in person-centred, reablement approaches.

A reablement approach works with people with dementia to augment as much as possible their functional and psychosocial capacity and independence. Reablement approaches to care, also known as ‘restorative care’, refer to maximising the health and wellbeing of older people through helping them participate in their daily, physical, social and community activities. When care and support are based on reablement, providers and practitioners collaborate and encourage the person to learn, restore and regain their functional and psychosocial capacity and independence as best as they are able [13, 14]. Research evidence in the non-dementia literature suggests that reablement reduces the need for ongoing traditional home care, is cost-effective in the long term despite initial costs, and improves outcomes for service users [15, 16]. However, evidence for reablement for people with dementia is still limited, with two exceptions: Care of Persons with Dementia in their Environments program (COPE) in the US [17], and community-based occupational therapy in the Netherlands [18]. Both programs are occupational therapist driven, with COPE including a brief nursing assessment component, and both have shown improved self-care and mobility in the person with dementia, as well as enhanced carer wellbeing.

Although these models have shown great promise, they do not address pain, mood, and other more biological components that nurses may address. In addition, there is a gap in understanding models of care that: 1) address common multimorbidity issues and associated functional decline using comprehensive and interdisciplinary teamwork; 2) readily fit within current health and aged care service delivery (in Australia); and 3) are client directed and goal oriented, with the person being in the centre [19].

### Theoretical framework of I-HARP

**Fig. 1:**
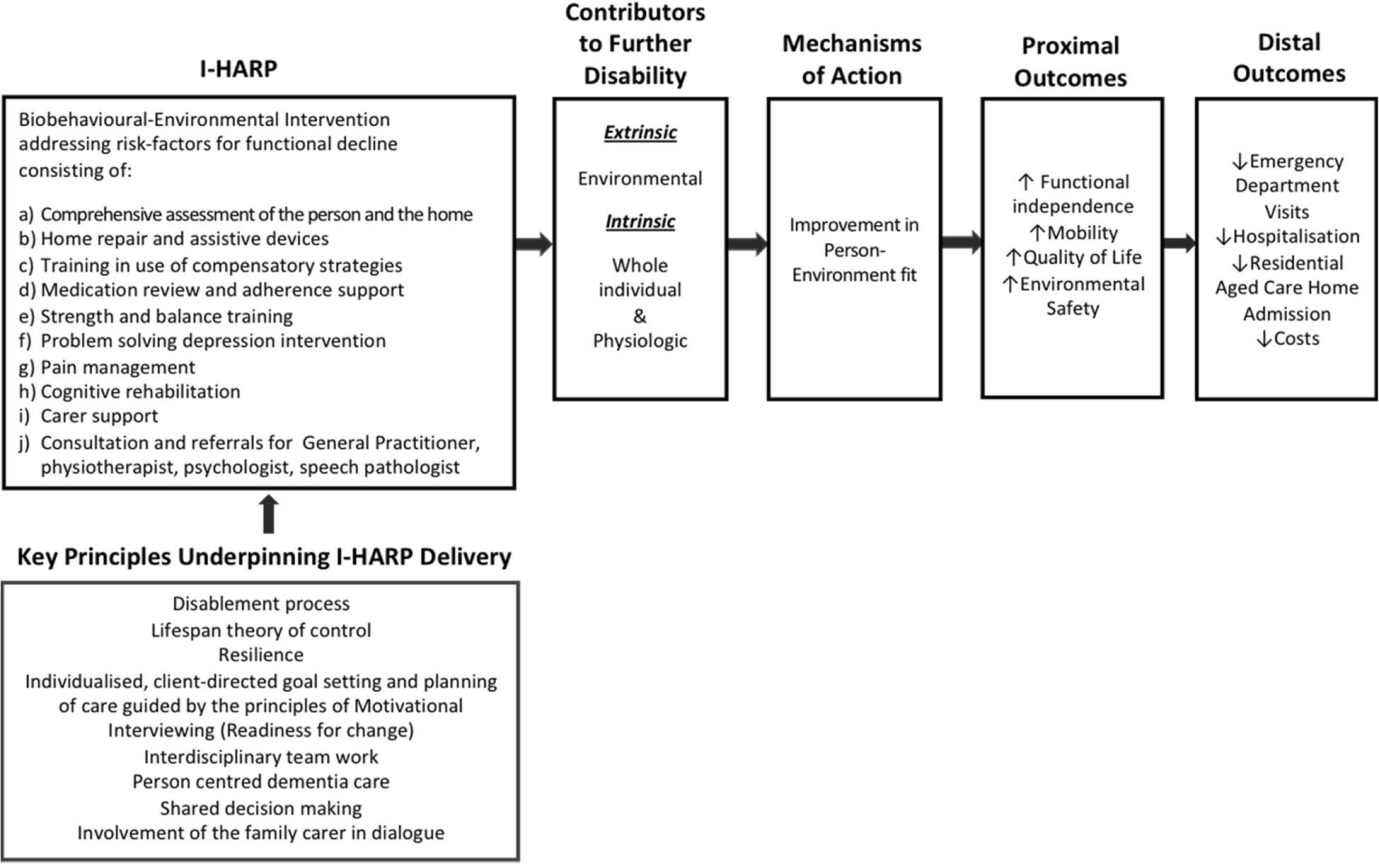
Program Logic for Interdisciplinary Home-bAsed Reablement Program (I-HARP). Note: The I-HARP program logic is a version of the Szanton et al. CAPABLE model [22, 23], modified to address the needs of people with dementia

I-HARP is an adaptation and expansion of the US reablement program Community Aging in Place Advancing Better Living for Elders (CAPABLE) [20–23], which accounts for common challenges that frail older people often experience concerning environmental risks for disability, functional decline and multimorbidity. As shown in Fig. 1, the CAPABLE framework uses sound theoretical and practice approaches, including person-environment fit theory [24], disablement processes [25], and lifespan theory of control [26] and resilience [27]. Practice approaches use individualised, client-directed goal setting and care planning, guided by the principles of motivational interviewing and interdisciplinary team work [20–23]. Further, tailored to the unique needs of people with dementia, I-HARP additionally incorporates the principles of cognitive rehabilitation, comprehensive cognitive and functional assessment, person-centred dementia care, shared decision making, partnership with the carer, and carer support.

Cognitive rehabilitation refers to “an individualised approach to helping people with cognitive impairments, by which those affected, and their families, work together with health care professionals to identify personally relevant goals and devise strategies for addressing these” [28](p.7). Cognitive rehabilitation has high ecological validity as it takes place in the client’s own environment and real-life context, and focuses on improving their everyday activities through “optimising residual cognitive abilities in impaired domains and making the most of unimpaired cognitive abilities” [28](p.8). A recent Cochrane review [28] concludes that evidence of cognitive rehabilitation is promising, particularly for people with mild dementia, in improving short and medium term outcomes such as competency and satisfaction in personal goal setting, memory and quality of life. Notably, our I-HARP pilot has strongly suggested that the effects of cognitive rehabilitation when combined with other multidisciplinary interventions can be extended to people with moderate dementia.

### Pilot work and feasibility of the I-HARP trial

In 2017, we completed a pilot RCT of I-HARP with community dwelling people with amnestic mild cognitive impairment (MCI) and mild to moderate stages of dementia [29]. The pilot showed the adequacy and appropriateness of the study design and procedures for randomisation, screening, recruitment and consent (19/25 eligible dyads consented), and adherence to the program (9/9 completed and complied), resulting in a total of 18 dyads in the trial. At 4 months post intervention and in comparison to a ‘usual care’ control group, I-HARP showed strong results in terms of goal attainment, improved mobility and independence, continued living at home with no entry to higher care, and both self-perceived and observed client’s wellbeing and confidence. The intervention group showed an improvement in functional independence while the control group (usual care) had a decline. The acceptance and benefits of I-HARP from qualitative interviews with carers and clinician field notes suggested a strong endorsement of I-HARP from most participants. Specifically, carers indicated a better understanding of how health issues affect everyday life activities, and reported the person becoming more independent and socially engaged as a result of I-HARP. Reasons for success were: one-on-one, hands-on approach; establishment of good relationships between I-HARP clinicians and clients; positivity and reassurance; continuity and regularity of visits; taking sufficient time with visits; trusting clinicians’ judgement; and specialised, yet easy to follow suggestions from each clinician. Some of the clients also said I-HARP gave them more confidence and physical strength. Prior to and during the I-HARP pilot the research team held multiple meetings and consultation sessions with consumers and service providers of aged/dementia care to seek their input into the I-HARP design, recruitment strategy and implementation in the Sydney metropolitan area. This process took over 6 months but proved to be necessary and crucial to the program refinement and success of the I-HARP pilot.

Despite their positive experiences, the carers in the intervention group showed some increase in burden, warranting further evaluation [30]. The current program has now been adapted and refined by engagement with consumer advocates, aged care providers and policy advisors, clinicians and researchers. Based on their feedback and the pilot findings, two additional components are now added to this larger trial: stronger carer support (3 carer sessions, instead of 1.5) and additional allied health support including dietitian, speech therapist, psychologist and physiotherapist.

In summary, a major gap exists in models of care provision for people with dementia who also experience multiple co-morbid conditions where multi- and inter-disciplinary team efforts would likely have higher impact [31]. I-HARP addresses this gap by integrating proven strategies into a comprehensive, person-centred, home-based, interdisciplinary intervention over 4 months with a goal to enhance the day-to-day function of older persons with dementia and other co-morbid chronic age-related conditions (e.g. pain, incontinence, polypharmacy, chronic illness). Notably, I-HARP closely aligns with the recommendations about *Promoting functional independence* (EBR67 & 68) in the Clinical Practice Guidelines [12]. Low levels of evidence for these recommendations support the need to generate stronger evidence in this area.

Two overarching aims of this project are: 1) to determine the effectiveness of I-HARP on functional independence, mobility, quality of life and depression among people with dementia, their home environmental safety, carer burden and quality of life, and I-HARP cost-effectiveness; and 2) to evaluate the processes, outcomes and influencing factors of the I-HARP implementation to inform consumers, practitioners, service providers and policy makers in their decision making and the use/delivery of the program, as well as to influence future systems-thinking and changes for dementia care.

### Methods: overarching design and conceptual frameworks

The overarching design is a mixed-methods action research approach, consisting of a multi-centre, pragmatic, parallel-arm randomised controlled trial, case audits and qualitative methods, conducted in two phases.

#### Phase I: a multicentre pragmatic parallel-arm stratified randomised trial

A multicentre pragmatic stratified RCT is being conducted to determine the effectiveness of I-HARP on daily activities, mobility, quality of life and depression, home environmental safety, and carer burden and quality of life, as well as I-HARP cost-effectiveness. The Advisory Group has been established, consisting of aged care peak organisations (Aged and Community Services, Leading Age Services Australia), aged care service providers (Community Care Northern Beaches, Whiddon Group, Prince of Wales Hospital Aged Care Service), consumer peak organisations (COTA, Dementia Australia), professional organisations (Australian College of Nursing, Occupational Therapy Australia), I-HARP research partner organisations (BaptistCare, Anglicare, Concord/Canterbury Hospitals, Royal North Shore Hospital), Northern Sydney Primary Health Networks (PHNs), and four consumers (people living with dementia and carers). The research team has worked closely with the Advisory Group to finalise the implementation plan for I-HARP. Adoption of I-HARP requires behaviour change by those involved (clients, carers, clinicians, service providers) and our implementation strategies are guided by Michie’s Behaviour Change Wheel (the COM-B theory): a ‘Behaviour system’ involving three essential conditions of Capability (physical, psychological), Opportunity (physical, social), and Motivation (reflective, automatic) [32]. We consult with the Advisory Group in tailoring our strategies and procedures for each site (e.g. appointing an I-HARP champion, coordinator and clinicians, and clinician training), detailing evaluation methods and processes (what and how best they can be implemented) as well as preparation for relevant ethics approvals. Training sessions for I-HARP clinicians, case coordinators and assessors involve structured group sessions led by study chief investigators (YHJ, RW, LM) and I-HARP trainers (RN and OT) who were involved in the pilot study.

Aligned with the I-HARP theoretical framework in Fig. 1, our hypotheses (HP) are that, compared to the usual care control group, the I-HARP group will have:

- At 20 weeks:
  - HP1 improved functional independence (primary outcome);
  - HP2 enhanced quality of life;
  - HP3 improved mobility;
  - HP4 reduction in depressive symptoms;
  - HP5 improved carer quality of life;
  - HP6 decreased carer burden; and HP7 improved home environment safety.
- At 52 weeks:
  - HP8 the benefits (HP1-HP7) will be sustained; and
  - HP9 I-HARP will lead to decreased total health care costs.

#### Phase II: evaluation of implementation processes

In Phase II of the study we evaluate the implementation strategies developed in Phase I, and the processes and outcomes of the I-HARP implementation using mixed methods. We aim to examine eight indicators of implementation success that are distinct from service and clinical effectiveness, including: Acceptability, Adoption, Appropriateness, Costs, Feasibility, Fidelity, Coverage/ Penetration and Sustainability. [33, 34]

In addition, we are exploring further research questions at both the individual and organisational levels, guided by a realist approach [35]. Realist evaluation is mostly suitable for: 1) new initiatives, or programs that seem to work but ‘for whom and how’ is not yet understood; and 2) programs that will be scaled up, to understand how to adapt the intervention to new contexts [36]. The research questions for Phase II include: 1) What changes did the clients and family and the I-HARP clinicians see during/after the intervention?; 2) Why did it work for some clients and not for others (for whom, in what context and how?) and for whom was the intervention most effective? (e.g., clinical and demographical factors, carer commitment, social capital, confidence and motivation, trust, acceptance and compliance, and environment)?; 3) What organisational and service factors influenced the implementation processes and outcomes of I-HARP (e.g., hospital based vs. community services)?; 4) What adaptations did the organisations have to make for the I-HARP implementation?; and 5) Which organisations are mostly likely to deliver I-HARP sustainably in the future?

### Methods: study sites, participants, intervention, outcomes and randomisation

#### Sites and sample size

We are recruiting participants across three public hospitals (two of the hospitals are combined into one research site as they have a common geriatric service) and two community aged care providers in the Sydney metropolitan area, New South Wales, Australia. A sample of 128 dyads will give 80% power at the 2-sided 5% significance level to detect a medium effect size of 0.5 between control and intervention groups on the primary outcome assessed at Time 2 (20 weeks, short term effect) and Time 3 (52 weeks, longer term effect). An effect size of 0.5 is clinically relevant as it implies that a trained observer can detect a difference between a control and intervention group in a specified outcome [37], in our case functional independence using the Disability Assessment for Dementia (DAD). We are confident that it is achievable as our pilot study showed a larger effect size of 0.61, as did a similar study of CAPABLE [21]. To allow for 25% loss to follow-up, 170 eligible dyads will be recruited. Based on consultations with the research site partner organisations, we estimated over 500 clients would be potentially eligible for I-HARP from the 4 research sites over 6 months, and a 35% participation rate (based on our previous community trials) would provide 175 dyads. However, current recruitment rates suggest an extra 12 months will be needed to meet the target sample size.

#### Participants

##### Inclusion criteria

Clients must:

1. be recipients of care and service from participating research sites;
2. have mild to moderate dementia rated on the Global Deterioration Rating Scale for Assessment of Primary Degenerative Dementia (GDRS) Stage 4–5 (mild-moderate) [2];
3. be aged 60 years or over;
4. have conversational English;
5. have a cognitively able carer who has at least 4 days or 7 hours per week contact;
6. consent to study participation; and
7. agree to be randomised.

Carers must also have conversational English and consent to study participation and randomisation processes.

##### Exclusion criteria

Clients will be ineligible to participate if they:

1. have a terminal illness with < 1 year expected survival or are having active cancer therapy;
2. plan to move in < 1 year;
3. are on a cholinesterase inhibitor, but have not been on a stable dose for at least 3 months);
4. have severe dementia (GDRS > 5);
5. have a home environment that is deemed unsafe for the I-HARP clinicians and assessors to carry out home visits (following pre-home visit safety screening); or.
6. are enrolled in another similar intervention trial to I-HARP.

Carers will be ineligible to participate if they have a moderate to severe level of cognitive impairment.

#### Recruitment

Participants are recruited with their carers from 1) a pool of clients eligible for federal government supported aged home care programs from the two participating aged care providers, and 2) patients from the three participating hospital geriatric services. To promote the recruitment process, the project team work closely together with the research site champions and the members of the Advisory Group who can advertise the study through their networks. The project team members carefully monitor the recruitment process and use active recruitment strategies such as public forums and local media to achieve recruitment targets.

#### Intervention and control groups

*Intervention group participants* receive I-HARP, which is a 4-month interdisciplinary model of care integrated with community aged care services, and hospital-based community geriatric services. The I-HARP intervention consists of the following components:

1. 12 home visits of 1.5 h (5–6 Occupational Therapy (OT), 3–4 Registered Nurse (RN), plus 2–4 additional options of a physiotherapist, speech pathologist or psychologist), tailored to the individual client’s needs.
2. Minor home modification and/or assistive devices up to the value of A$1000.
3. Three individual carer support sessions of 1.5 h at the beginning, middle and end of home visits, conducted by a case coordinator.

I-HARP interventionists receive on-going supervision and mentoring support in delivering the intervention and their performance is periodically reviewed with quality checks.

Home visits by the I-HARP clinician include:

1. An initial comprehensive assessment performed by a) an OT for cognitive and functional abilities, strength, balance and home safety risks, and b) an RN for the medication regimen, pain, incontinence, depression and other co-morbid and chronic disease management issues.
2. Subsequent interdisciplinary, tailored care planning to enhance self-care ability using person-centred goal setting. I-HARP clinicians work closely with the person with dementia and their carer to help them identify goals and action strategies that the client aims to achieve in the following 3 months, using the Bangor-Goal Setting Interview.
3. Implementation of the plan through a series of home visits, including cognitive rehabilitation, combining compensatory (e.g. calendars, diaries, reminders) and restorative strategies (e.g. mnemonics, semantic association, spaced retrieval), energy conservation and task simplification strategies, balance and strength exercises, pain relief, anxiety and depression management, problem solving, medication simplification/adherence training, and minor home alterations and assistive devices.

The carer support sessions are individually tailored and delivered by an experienced facilitator (I-HARP case coordinator). The sessions cover dementia and its impact, principles of reablement and person-centred care, the goals of I-HARP, carer’s role in I-HARP, as well as discussing the carer’s needs and concerns (e.g. self-care, communication, behaviours of concern, accessing services, powers of attorney, advance care plans, enabling the person, and any issues arising from I-HARP).

The findings of the pilot showed that success of the intervention was highly influenced by the carer’s understanding of the person’s capabilities and need for reablement approaches to care, as well as their partnership with the I-HARP clinicians. Emphasis on carer education on these aspects during the carer sessions and throughout the I-HARP clinicians’ interactions with the carer, has been an important component of the training. An important part of I-HARP is interdisciplinary teamwork and coordination of the program, facilitated by a face-to-face case conference meeting after the initial assessments. Based on the initial assessment, the need for other allied health professional involvement (physiotherapist, speech pathologist and/or psychologist) is determined. If required, the allied health professional works closely with the OT and RN. Fortnightly emails and phone conversations between all clinicians occur throughout the duration of the program. The I-HARP clinicians also ensure that the family carer is actively and meaningfully engaged in all processes as well as possible.

**Fig. 2:**
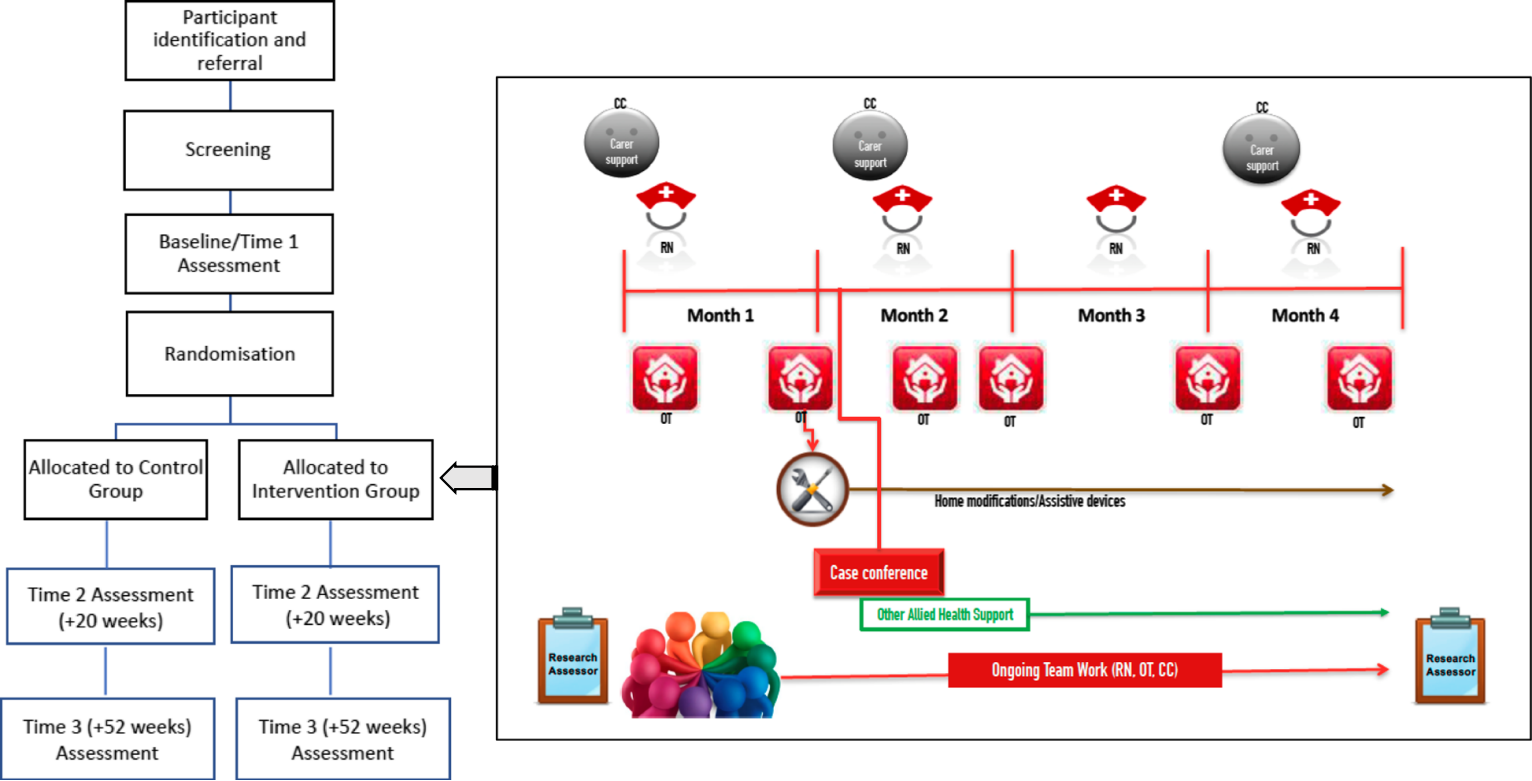
Study flow chart with a pictorial illustration of the I-HARP Delivery. CC: Case Coordinator, OT: Occupational Therapist, RN: Registered Nurse

Figure 2 provides a pictorial illustration of the I-HARP delivery.

*The control group participants* receive a set of two movie vouchers (per dyad) every three months, offered based on ethical grounds, so that participants receive some benefit from taking part in the study, without influencing the outcomes. They are allowed to receive usual care under their hospital- or community-based aged care services, which may involve ad hoc nursing and allied health services, and home modifications, without the components of cognitive rehabilitation, or structured carer support.

#### Assessment

There are three assessments: Baseline/Time 1, prior to intervention; Time 2, 20 weeks post baseline; and Time 3, 52 weeks post baseline. All assessments are being conducted by an experienced clinician blinded to group allocation.

*Baseline measures* include socio-demographic data (age, sex, ethnicity, pension status, health insurance, education) and clinical data (health and cognitive conditions, medications). The Addenbrooke’s Cognitive Examination Third edition (ACE-III) [38] and the GDRS [2] are being used to record cognitive function and dementia severity. Following baseline assessment, participants are randomised to either the intervention or the control group.

*Outcome measures* consist of primary and secondary outcomes. The primary outcome is the client’s functional independence at Time 2, assessed using the Disability Assessment for Dementia (DAD) [1] which measures self-care disability and independent living skills. Secondary outcomes are: self-care disability and independence at Time 3; both short- and long-term effects on mobility and physical function, health-related quality of life, carer burden, carer quality of life, and home environmental safety; and total health care costs over the 52 weeks following randomisation. The secondary outcome measures are:

- Short Physical Performance Battery (SPPB) [39] for objective physical function
- Collateral Source version of the Geriatric Depression Scale-15 item (CS-GDS-15) [40] for depression
- Quality of life in Alzheimer’s disease (QOL-AD) [41, 42] for general quality of life
- 5-Level version of the EuroQol five dimensions (EQ-5D-5 L) [43, 44] for health-related quality of life (both client and carer) for the economic evaluation, using the Australian weighting algorithm [45]
- Zarit Burden Inventory (ZBI) [46] for carer burden
- The Home Safety Self-Assessment Tool (HSSAT) for the overall home environment safety.

In addition, monthly telephone contact is made with all carers to monitor participants’ use of prescribed medicines and costs associated, healthcare and community services (type, duration and frequency, and personal costs), incidents of falls and minor injuries as well as carers’ workforce participation (i.e. total number of work hours in the last week). To ensure accurate recording, carers are given a diary/log to complete and during the monthly phone call this is verified. This method was used successfully in the I-HARP pilot.

##### Implementation process evaluation

Multiple sources of data are being collected including:

1. case/clinical notes and field notes from I-HARP clinicians and case coordinators;
2. I-HARP related administrative data and case audit for each of the four research sites;
3. interviews with all consenting study participants after Times 2 and 3 data collection, providing further accounts of their experiences with reablement approaches to care either as I-HARP participants or as control group participants in their usual care (n ≈ 100); and
4. focus group interviews with I-HARP interventionists and key managers of the participating research sites after Time 2 data collection, providing perspectives of the participating organisations and interventionists on reablement approaches to care for people living with dementia and I-HARP processes, and mechanisms of their decision making (4 focus groups, 1 per site, with 4–6 people per group).

All interviews are conducted in a combination of semi-structured and structured form designed specifically for the I-HARP evaluation. Both clients and carers are invited to be interviewed individually. The interviews with study participants take about 45–60 min and are conducted face-to-face at home or via telephone, depending on their preference. The focus group interviews will take about 1–1.5 h and be conducted face-to-face at each participating research site. For the focus groups, the participants will be given opportunity to provide further comments to the research team if they have not been able to voice their concerns or issues during the group discussion.

#### Randomisation, allocation concealment, and blinding

After recruitment to the study and collection of baseline data (Time 1), each participant is randomly allocated (1:1) to either the intervention or control arm. Randomisation is performed separately for each site and stratified by severity of dementia (mild vs. moderate). Randomisation is performed by computer-generated random permuted blocks of varying size provided by JMS [47]. Opaque sequentially numbered envelopes are used to ensure allocation concealment. Participants are not blinded to group allocation, but assessors and statisticians are. Participants are notified by phone and the importance of blinding is explained. Ethics principles are applied to ensure that participants are supported in this process and understand the blinding processes and potential consequences. The members of the research team responsible for data collection from people with dementia and carers, data entry and analysis will remain blind until completion of the main analysis. Unblinding is permissible for the person who is collecting a monthly carer diary as it may contain information about I-HARP service. This will not contaminate the data to be collected as the information is supplied by the carer.

### Methods: data collection, management, analysis, and quality assurance

#### Data collection and management

As shown in Fig. 2, study outcome measure data are collected at 3 assessment times: Baseline /Time 1 at 0 weeks, Time 2 at 20 weeks and Time 3 at 52 weeks. Data are managed using a REDCap electronic database, which is a secure, web-based application designed to support data capture for research studies. The system provides: 1) an intuitive interface for validated data entry; 2) audit trails for tracking data manipulation and export procedures; 3) automated export procedures for seamless data downloads to common statistical packages; and 4) procedures for importing data from external sources. All electronic data are stored in password-protected, secure computer servers/systems and paper records are stored in locked filing cabinets in access-controlled facilities. All data entries are completed via a password-protected validated encrypted study electronic case report form. Only de-identified information is uploaded to the database.

Information collected during the delivery of the intervention (e.g., case notes, case conference notes, audio records of interventions, completed visit checklists) are uploaded to the University of Sydney endorsed secure website. Each site is allocated their own secure folder for use and the interventionists are given clear instructions not to enter any participant’s personal information and to refer to participants only using their participant ID in all their communications and when transcribing information. The project team ensures that the privacy, security and ownership of the research data is maintained, and the data will not be stored or accessible by another organisation without prior ethics approval.

In compliance with the NSW State Records Act, the archiving period for clinical research records will be 15 years (NSW supplement to the National Statement, section 3.3.11). At the conclusion of the archiving period we will consult with the University of Sydney Archives and Records Management Service for advice on the most appropriate manner of destruction and disposal of the research data. Further details about data security and management can be provided by the principal investigator (YHJ) on request.

#### Data analysis plan

Each indicator of the implementation outcomes will be analysed using descriptive and inferential statistics as well as qualitative content analysis. All analyses of RCT outcomes will be by intention-to-treat (i.e. including all participants, as randomised, regardless of protocol adherence). The primary analysis of the primary outcome (using the DAD) and secondary outcomes will be an unadjusted comparison between intervention and control groups at Time 2 (short-term effect) and Time 3 (longer-term effect) using two sample t-tests of the change from baseline, or Mann-Whitney tests for outcomes that are not normally distributed. Secondary analyses will use linear regression to adjust for the baseline value of that outcome, stratification variables (site and severity of cognitive impairment) and other covariates (age and pension status).

Qualitative field notes and interview data will be fully transcribed and entered into NVivo 11 (qualitative data analysis software package) for analysis. Two-stage data analysis will be conducted: analysis of the field notes and study findings (quantitative data) followed by qualitative content analysis of interviews with research participants (n ≈ 100) and I-HARP interventionists and participating site managers (*n* = 16–24) to explore their perspectives on reablement approaches to care and I-HARP processes, and mechanisms of their decision making.

The economic evaluation of I-HARP involves costing the intervention itself (e.g. clinicians’ training time, delivery, travel, supervision, care coordination time, minor home modification/assistive devices, intervention materials) and any change in carer workforce participation and health-related client costs over the 52-week period (medications, allied health services, community/aged care, visits to specialists, GPs, hospitals). By combining these cost data with outcome data, relating to mortality and health-related quality of life (using the EQ-5D-5 L), a cost-utility analysis will be undertaken, reporting a cost per quality-adjusted life year (QALY) of the I-HARP intervention relative to controls. The economic evaluation will present both a ‘within-trial result’ (i.e. considering only those costs and outcomes accruing over 52 weeks) as well as to be extrapolated over a longer time period, such as ten years. As we will have 52 weeks of participant-level data, we will apply bootstrapping methods to estimate the uncertainty around cost-effectiveness figs. [48].

#### Retention

To promote participant retention and complete follow-up, the project team send out season’s greetings/birthday cards and a small gift (<A$10) to all participants; and follow participants to a nursing home to complete assessments if required. To accommodate for client hospitalisations for less than 2 months during the intervention period, the research team employ ‘stop the clock’ on the intervention until the client is back home or start over and re-do baseline when they get home as their cognitive and physical status may have changed post-hospitalisation. There is a possibility that due to cognitive decline clients will have dementia symptoms consistent with GDRS level 6 at this time. In these cases the clients would still continue their participation in the study because the intention to treat was present at time of enrolment. Decisions as to how many hospital readmissions, and ‘stop the clock’, are allowed per client will be determined on a case by case basis, through consultations with the trial investigators.

#### Data consistency and monitoring

Prior to the initiation of the study at any participating centre, all investigators and designated site personnel including I-HARP interventionists and clinical assessors were trained on the study procedures. The training for interventionists and assessors is standardised. Trial monitoring including all matters concerning participant assessments, data collection, intervention delivery, record keeping, and data entry, is performed by the I-HARP data monitoring team on an on-going basis. Accuracy of data entry (100% of the primary outcome measure and 20% of baseline and secondary outcome measure entries) and 100% of the participant consent forms are checked by the data monitoring team. Regular site visits are carried out to ensure that the study is conducted according to ICH-GCP guidelines and as per protocol requirements.

An independent Data Safety and Monitoring Committee (DSMC) was established during Phase I, which meets twice a year to monitor the quality of trial data and the safety of research participants. DSMC membership consists of experts in clinical trial conduct, statistics, aged care and dementia, and terms of reference were determined during the first month of the project. The study is subject to a random audit by the University of Sydney. Any source information and other study files must be accessible at all study sites at the time of auditing and inspection during the course of the study and after the completion of the study.

#### Adverse/serious adverse events

All adverse events and serious adverse events are reported to relevant authorities as per the principles of the *National Health and Medical Research Council Guidance: Safety monitoring and reporting in clinical trials involving therapeutic goods* [49]. This information will also be reported in the annual progress report to the relevant ethics committee and reports to the DSMC.

#### Fidelity

The treatment fidelity plan is based on the CAPABLE Trial Feasibility Strategies [22, 23] which were informed by the NIH Behaviour Change Consortium [50]. The study planning and design has incorporated several steps to ensure fidelity. For example, the site personnel who deliver the intervention have been selected carefully to ensure that they have the necessary qualifications, skills and experience to deliver the intervention to the expected standard. I-HARP clinicians require a minimum 2 years’ experience in the field and person-centred care practice. Group training sessions before the commencement of the study and refresher sessions have been held for I-HARP interventionists from all sites. They have been provided with training manuals and a checklist and trained in assessment tools and intervention techniques. Collection of field notes and case notes is monitored regularly by the project team. The I-HARP checklist, detailed session notes of what has been planned and achieved for each home visit, and 20% of the audio recorded case conferences and home visit sessions are being randomly selected and reviewed by the project team for quality check. The delivery of the interventions and participant compliance with the intervention are also monitored.

Please refer to Table 1: The schedule of enrolment, interventions, and assessments, outlining the timeline of the trial procedures.

**Table 1:** The schedule of enrolment, interventions, and assessments

TIMEPOINT^b^	STUDY PERIOD						
	Enrolment	Allocation	Post-allocation				Close-out
	T_1a_ *0 week*	T_1b_ *0 week*	T_2a_ *for 20 weeks*	T_2b_ *at + 20 weeks*	T_3a_ *for 52 weeks*	T_3b_ *at + 52 weeks*	T_4_ *6 months from* T_3b_
ENROLMENT:							
Eligibility screen	X						
Informed consent	X						
Randomisation & Allocation		X					
INTERVENTIONS:							
I-HARP intervention			X		–		
Control: Care as usual			–		–		
ASSESSMENTS:							
*Baseline Variables*							
Global Deterioration Rating Scale (GDRS)	X						
Socio-demographic and basic clinical data	X						
Addenbrooke’s Cognitive Examination (ACE-III)	X			X		X	
*Outcome variables*							
Disability Assessment for Dementia (DAD)	X			X		X	
Short Physical Performance Battery (SPPB)	X			X		X	
Collateral Source version Geriatric Depression Scale (CS-GDS)-15	X			X		X	
Quality of life in Alzheimer’s disease (QOL-AD)	X			X		X	
EuroQol 5 dimensions (EQ-5D-5 L) (client and carer)	X			X		X	
Zarit Burden Interview (ZBI)	X			X		X	
Home Safety Self-Assessment Tool (HSSAT)	X			X		X	
*Other Data Variables*							
Carer diary monthly	X		X	X	X	X	
Interviews with participants				X			
Focus groups with key stakeholders						X	
Adverse/Serious adverse event assessment^a^			X	X	X	X	
Fidelity check	X	X	X	X	X	X	
Reporting of study results^b^							X

^a^Adverse events are monitored and reported as they occur, for the duration of the study

^b^We expect to take about 6 months to complete data analysis and compile the final results paper for an appropriate journal. A summary of the study results will be made available to the referring clinicians and service providers, and study participants

### Dissemination policy

A variety of communication activities will be timed to align with the achievement of key project milestones, targeting different audiences. Key examples are listed below.

- Producing I-HARP e-newsletters at least twice a year for the promotion and updates of I-HARP among stakeholders (consumers, community groups, service providers);
- Forwarding of newsletters to promote I-HARP through dementia and aged care related media including Australian Ageing Agenda, Talking Aged Care, Dementia Australia and *Primary Health Network and aged care peak body newsletters;*
- Peer reviewed publications reporting study results, editorials and letters to the editor; and
- Showcasing I-HARP on the Aged Care Channel and presentations at local and international conferences and webinars, as well as consumer seminars and events.

I-HARP participants will be provided with a three-page summary of the study findings at the conclusion of the study. Authorship for peer reviewed publications will be based on the International Committee of Medical Journal Editors Guidelines and Recommendations. http://www.icmje.org/

### Trial sponsor

The University of Sydney.

Email: clinical-trials.research@sydney.edu.au

## Discussion

A major gap exists in Australia and internationally in providing care to support and maintain functional and social independence of older people with dementia living at home. Psychological and neurological impairments in people with dementia have negative consequences for their functional and social participation and limit the person’s everyday living and experiences. People with dementia are likely to have difficulties with independently preparing meals, managing finances, making phone calls, shopping, taking medications, dressing, showering and toileting, but critically, they retain the capacity to enjoy a meaningful life with the most appropriate care [51]. In order for people to live well with their dementia, key services and best care practices are needed that recognise and maximise their capacity and capabilities to engage in their daily, physical, social and community activities. I-HARP addresses one of the costliest, often overlooked and significantly undertreated, aspects of old age, particularly among people with dementia: the ability to carry out every-day self-care activities and maintain independence.

Despite mounting evidence concerning reablement approaches to care and the need for interdisciplinary services, no dementia-specific, interdisciplinary reablement model of care has been rigorously trialled in Australia. I-HARP builds on the USA’s CAPABLE study by focusing on people with dementia and working in partnership with family carers. As demonstrated in our pilot, we hope that the key benefits of I-HARP will include the person’s decreased disability in self-care and improved independent living skills, enhanced health-related quality of life, improved functional capacity and confidence in doing activities without falling. It further has potential for reduction in depressive symptoms and care burden, improved carer quality of life and potentially decreased total health care costs in the long term. The I-HARP pilot has also shown that intervention from an interdisciplinary team has the maximum impact. Successful implementation of I-HARP will thus contribute to the following aspects of dementia care and service delivery: early intervention and management; support for people with dementia and their carers in the community that encourages personhood, choice and personalised goal setting.

This project focuses on the implementation and evaluation of I-HARP in existing hospital-based community programs and community aged care settings. The overarching design is a mixed-methods action research approach consisting of a multi-centre, pragmatic, parallel-arm randomised trial, case audits and qualitative methods. During the final phase of the study we will develop future strategies and directions for promotion, dissemination, implementation, and evaluation and sustainability. The research team and Advisory Group will use the information collected on I-HARP performance and processes to make recommendations for improvements and sustainability, and to cite the successes of the I-HARP project. In particular, we will investigate how I-HARP could be incorporated into existing hospital-based geriatric services or home-care programs (Commonwealth Home Support Programs, Home Care Packages, Short Term Restorative Care Program).

This design will allow us to generate evidence for the effectiveness of I-HARP as well as investigate the processes, outcomes and influencing factors of the program implementation to inform consumers, practitioners, service providers and policy makers in their decision making. Implementing a new model of care requires meaningful collaboration between all parties towards change; active engagement and empowerment are two of the most important elements to success. Action research enables this process as it focuses on finding solutions to practical concerns through collaborative, democratic and emancipatory processes [52]. There are four broad rationales for using this evaluation method: 1) to increase accountability, 2) to “steer” the research process, 3) to provide a means for “advocacy”, and 4) to provide an input into the management improvement process (through better understanding and learning). The mixed method action research approach has been used successfully by the investigators in previous studies on models of care and aged care leadership development [53, 54] and found to be effective as it facilitates active involvement of and collaboration between the researchers and key stakeholders in the cycles of change; from project design, problem solving, delivery of research, and planning through to project acceptance and successful implementation, evaluation and translation of project outcomes.

Another strength of the project is co-design of the study with consumers and their involvement throughout the implementation and evaluation processes. Participant consumer involvement is particularly important to the “real-world” significance of this project, as people living with dementia and their carers or family members can surely provide the most relevant insight into real-life problems and needs for this target group. In this project, consumer participants are engaged in two ways:

- They are invited to join the Advisory Group which will provide a platform for provision of insight and suggestions for people living with dementia and their family/carers. This informs the final planning of I-HARP at Phase I and will contribute to the evaluation of the program. They will participate in the project via a combination of face-to-face meetings, email correspondence and video conferencing to provide their unique perspective on the project’s plan, implementation, dissemination and translation strategies at the individual level.
- They are involved as research participants, who consistently provide feedback during the face-to-face intervention sessions (as I-HARP interventionists’ field notes form part of the trial’s evaluation methods) and post intervention in-depth interviews (carers), which will provide further opportunity for invaluable comments on the utility of the program for each dyad, with respect to the specific goals of reablement and person-centred care.

Furthermore, establishing broader consumer and community engagement is another important element of implementation evaluation. In order to ensure I-HARP is readily incorporated into the existing home-care programs or hospital-based geriatric services, is scalable and produces optimal outcomes, we are testing I-HARP in the aged care and health care systems – e.g. community aged care services operating under Commonwealth Home Support Programs (CHSP) and Home Care Packages (HCP), and hospital-based community geriatric services, which are designed to support frail community-dwelling older people to maximise their independence in their home environment. As part of the aged care reforms, the Australian government has introduced a number of measures and policies in the provision of care and support for older Australians including changes in CHSP and HCP, and the recently introduced Short Term Restorative Care (STRC) program. Furthermore, with the full implementation of the Consumer Directed Care (CDC) and funding for the CDC packages directly allocated to and following the consumer, patients are given greater control over their choices about not only the home care provider, but also the types of care and services they receive and how those services are delivered. Such contextual information will be taken into consideration in the development of future strategies and directions for promotion, dissemination, implementation, evaluation and sustainability.

I-HARP integrates evidence-based strategies into a dementia-specific person-centred, time-limited, home-based, interdisciplinary rehabilitation package. The project implements and evaluates this novel bio-behavioural-environmental I-HARP model, integrated into existing health and aged care services. The trial will confirm I-HARP’s scalability in community aged care services operating under Commonwealth supported home care service, and hospital-based geriatric services, both of which are designed to support frail community dwelling older people to maximise their independence in their home environment. The Australian government has pledged to deliver “timely, high quality entry-level support services taking into account each person’s individual goals, preferences and choices – and underpinned by a strong emphasis on wellness and reablement” [55] (p.3). Our mixed methods action research approach, combined with a full-scale pragmatic RCT and realist evaluation, will provide a comprehensive picture of the impact, and factors contributing to the outcomes of the intervention, as well as evidence of whether or not I-HARP is cost effective in the real-life context. Such timely insights will provide policy makers and service providers with information relevant to the roll-out of the program in the wider community.

## Data Availability

The datasets used and/or analysed during the current study will be available from the corresponding author on reasonable request.

## Abbreviations

ACE-III: Addenbrooke’s Cognitive Examination-III
ADL: Activities of Daily Living
CAPABLE: Community Aging in Place Advancing Better Living for Elders
COPE: Care of Persons with dementia in their Environments program
CS-GDS-15: Collateral Source version Geriatric Depression Scale-15 item
DAD: Disability Assessment for Dementia
DSMC: Data Safety and Monitoring Committee
EQ-5D-5 L: 5-Level version of the EuroQol five dimensions
GDRS: Global Deterioration Rating Scale
HREC: Human Research Ethics Committee
HSSAT: Home Safety Self-Assessment Tool
ICH-GCP: International Conference on Harmonisation – Good Clinical Practice
I-HARP: Interdisciplinary Home-bAsed Reablement Program
NHMRC: National Health and Medical Research Council
OT: Occupational Therapist
QOL-AD: Quality of life in Alzheimer’s Disease
RN: Registered Nurse
SPPB: Short Physical Performance Battery
ZBI: Zarit Burden Interview
